# Brain Drain and Health Workforce Distortions in Mozambique

**DOI:** 10.1371/journal.pone.0035840

**Published:** 2012-04-27

**Authors:** Kenneth Sherr, Antonio Mussa, Baltazar Chilundo, Sarah Gimbel, James Pfeiffer, Amy Hagopian, Stephen Gloyd

**Affiliations:** 1 Department of Global Health, University of Washington, Seattle, Washington, United States of America; 2 Human Resources Department, Mozambique Ministry of Health, Maputo, Mozambique; 3 Eduardo Mondlane University, Maputo, Mozambique; Universidad Peruana Cayetano Heredia, Peru

## Abstract

**Introduction:**

Trained human resources are fundamental for well-functioning health systems, and the lack of health workers undermines public sector capacity to meet population health needs. While external brain drain from low and middle-income countries is well described, there is little understanding of the degree of internal brain drain, and how increases in health sector funding through global health initiatives may contribute to the outflow of health workers from the public sector to donor agencies, non-governmental organisations (NGOs), and the private sector.

**Methods:**

An observational study was conducted to estimate the degree of internal and external brain drain among Mozambican nationals qualifying from domestic and foreign medical schools between 1980–2006. Data were collected 26-months apart in 2008 and 2010, and included current employment status, employer, geographic location of employment, and main work duties.

**Results:**

Of 723 qualifying physicians between 1980–2006, 95.9% (693) were working full-time, including 71.1% (493) as clinicians, 20.5% (142) as health system managers, and 6.9% (48) as researchers/professors. 25.5% (181) of the sample had left the public sector, of which 62.4% (113) continued working in-country and 37.6% (68) emigrated from Mozambique. Of those cases of internal migration, 66.4% (75) worked for NGOs, 21.2% (24) for donor agencies, and 12.4% (14) in the private sector. Annual incidence of physician migration was estimated to be 3.7%, predominately to work in the growing NGO sector. An estimated 36.3% (41/113) of internal migration cases had previously held senior-level management positions in the public sector.

**Discussion:**

Internal migration is an important contributor to capital flight from the public sector, accounting for more cases of physician loss than external migration in Mozambique. Given the urgent need to strengthen public sector health systems, frank reflection by donors and NGOs is needed to assess how hiring practices may undermine the very systems they seek to strengthen.

## Introduction

The shortage of health workers in resource-limited settings is a well-established constraint to building sustainable, quality public sector health systems, and achieving improved health outcomes [1], [2]. Numerous factors contributing to the human resources for health crisis have been identified, including lack of sustained funding for production of new health workers [3] and macro-economic policies that cap the absorption of health workers into the public sector and hinder efforts to retain skilled health workers through limiting salaries and worsening working conditions [4]. External “brain drain”, or migration of trained health workers to work in wealthier countries for higher salaries, continues to plague resource-limited settings [5], [6]. However, internal migration, or the movement of health workers from the public sector to work for other entities within the same country including non-governmental organizations (NGOs), the private sector, and multi-lateral and bi-lateral donor agencies, has received less attention though it has an important impact on public sector capacity [7].

Over the last two decades international NGOs have played an increasingly important role in supporting public sector health systems in low and middle-income countries, fuelled largely by shifts in funding away from support for state health systems to NGOs as well as other private sector providers [8], [9], [10], [11]. Reports suggest that these actors compete with public sector health systems for local qualified staff [12], [13]. In recent years, the substantial increase in funding for health activities outside the public sector through vertical initiatives, such as PEPFAR and the Global Fund to Fight HIV, TB, and Malaria, has led to a rapid rise in NGO-directed funding, which has created conditions leading to accelerated internal migration.

Existing research on human capital migration has focused on documenting the extent of external migration, often through licensed health professional registration systems in middle and high-income countries. Research on internal migration remains limited [14], [15], [16], [17]. One study found that over half of the 52 graduates from Uganda’s Makerere University 1984 graduating class worked outside the public sector [18]. A study of 200 medical school graduates from South Africa showed high attrition from the public sector to the private sector [19]. Given the limited research in this area, the current emphasis on strengthening health systems to meet HIV/AIDS and Millennium Development Goals in poor countries, and the increased availability of external assistance to the health sector largely through NGOs, systematic research on internal brain drain is urgently needed [8], [20], [21], [22], [23].

This study documents the extent of both internal and external physician migration from the public sector in Mozambique among physicians qualifying from medical school between 1980–2006. We focus on physicians because they are the most influential leaders and managers in the Mozambique health system, and considering their limited numbers, have the most dramatic effect on health system functioning when they leave the public sector.

Mozambique provides an especially valuable case study for exploring brain drain, and particularly internal migration, from the public sector. Established after independence in 1975, the Mozambique National Health Service (NHS) is a strong, cohesive network providing the majority of formal health care in the country [24], [25]. During the first 15 years after independence, Mozambique experienced a protracted civil war in which few NGOs were active in the country. By the end of the war in the early 1990s NGOs began arriving in large numbers to support the NHS, though a few provided direct patient care. The NGO model has grown rapidly in recent years with increased vertical funding from traditional bilateral donors and global initiatives such as PEPFAR and the Global Fund that has been channeled mostly through NGOs (vertical funds is estimated to have reached $300 million, or 59 percent of health sector expenditure, in 2008, compared with $75 million, or 36 percent of health sector expenditure, in 2003) [26], [27]. Anecdotal and grey literature reports suggest that internal migration to NGOs and donor agencies has rapidly increased as vertical funding has grown. Furthermore, the small number of medical schools that feed graduates into the health sector allows for a comprehensive overview of physician loss from the public sector.

## Methods

### Ethics Statement

The research protocol was approved by the ethics review board of the Mozambique Ministry of Health. Written informed consent for study participants was obtained prior to being interviewed.

### Participant Selection and Description

A prospective study design was used to describe the career pathways for Mozambican physicians qualifying between 1980–2006. Data collection initiated with physicians qualifying in 1980 to capture career decisions most directly linked to public sector health system capacity; pre-1980 graduates were excluded as they initiated medical school before the creation of the NHS, and arguably had different professional objectives than those initiating medical school post-independence.

Because there is no central roster of Mozambique physicians approved to work for the NHS, we employed two methods to generate rosters of physicians by year of qualification. Annual graduation lists were used to identify graduates from the only Mozambican medical school with graduates before 2006 (Eduardo Mondlane University). Rosters of physicians qualifying from universities outside of Mozambique (predominantly Cuba, the former USSR, and Eastern Europe) were generated by interviewing over 15 graduates of these foreign institutions and 11 provincial human resource managers with access to individual personnel files. We believe this approach to generating graduation lists for foreign medical schools was extensive and sound given the informal networks that were created among Mozambicans trained in a limited number of universities. Based on discussions with health authorities and that no additional physicians were identified through the 2010 data collection exercise compared with the 2008 collection period, we estimate that the overall roster covers over 97 percent of the total number of Mozambican physicians trained between 1980–2006.

### Ascertainment of Employment Status

Between April-May, 2008, and again in July, 2010, up-to-date employment status for physicians was ascertained by interviewing three to eight physicians from each graduating year on the current employment situation of their classmates, as well as others who they may know either because they graduated over generally the same time period (two to three years before and after their qualification date), graduated from the same foreign training institution, or through working in the same health facility or province. These study informants were selected because they were known by the study investigators or were referred by their classmates.

Current employment status was ascertained for physicians working in the public sector by contacting the Ministry of Health’s (MOH) Human Resources Department in all 11 provinces, at the Central level, and the Maputo Central Hospital, where individual employment records and payment information are stored. Information for those working for NGOs or donor institutions were confirmed by contacting employees at these organizations. This data triangulation resulted in information on all physicians with the exception of five working outside the country for whom specifics on current employment could not be ascertained.

Information collected on each physician included current work status (working, studying full-time, not working due to illness or death, unemployed), institution of current employment (government in general and specifically which sector, NGO, bi-lateral or multi-lateral donor, private sector), geographic location of current employment (country, province, city), post-degree training (yes/no, and if yes, clinical and/or post-graduate certificate or degree in public health of at least one year in duration), and type of job (management, clinical, teaching, research, other). Type of job was defined by the primary duty of the position, such that facility or administrative unit directors were categorized as managers, staff physicians and chief medical officers as clinicians, training centre staff as educators, and those working for research institutions as researchers. Notably, physicians currently pursuing a full-time clinical specialty were categorized as performing a clinical function given that the majority of their time is spent attending patients in the clinical setting.

Our primary outcome measure was whether a physician worked in the public sector at the time of data collection. Physicians living and working outside of Mozambique were categorized as a case of external migration, while those working inside Mozambique, but no longer paid through the public sector, were categorized as a case of internal migration. Physicians who worked part-time in the private sector or doing short-term consultancies but whose principal role was within the public sector were categorized as public sector employees. Physicians who previously held a senior-level management position in the public sector were identified, including national positions (Ministers, Vice Ministers, National Directors, Adjunct National Directors, and Program Directors) and provincial positions (Provincial Directors, Chief Medical Officers, and Directors of one of the three quaternary-level referral hospitals).

Data analysis included descriptive statistics and unadjusted relative risk measures of association. Analysis of factors associated with physician migration was limited to physicians qualifying before 2003, as physicians graduating from 2003–2006 were generally in post-training rural service or specializing, and do not represent the experience of physicians who are more established in their careers. Annual incidence of physician migration was estimated by counting new cases of migration both into and out of the public sector over the 26-month period between the 2008 and 2010 data collection. Data were analyzed using Stata v10 (College Station, TX).

## Results

We identified a total of 723 Mozambican physicians graduating between 1980–2006. As of July, 2010, more than 30 percent of these physicians had completed a post-graduate degree, of which nearly three-quarters were a clinical specialty (Table 1). Eighty-five percent of the sample graduated from Eduardo Mondlane University, which was the only Mozambican medical school qualifying physicians until 2006, while nearly 13 percent were trained at medical schools in Cuba and the former USSR. Over 95 percent of graduates were working full-time as of July, 2010, including 17.8 percent (N = 123) who were pursuing a post-graduate degree in public health on a part-time basis (N = 13) or a clinical specialty (N = 110). Among physicians working or studying full-time in July, 2010, over 70 percent had predominantly clinical duties, while the remaining physicians had management, teaching, or research roles. Over 90 percent of physicians in the study were working in Mozambique as of July, 2010, nearly half in the capital city, Maputo.

**Table 1:** Characteristics of Mozambican medical school graduates between 1980–2006, as of 07/2010.

	N	(%)
Sex		
Female	366	(50.6)
Male	357	(49.4)
		
Post-graduate trained	234	(32.40)
		
Type of post-graduate training		
Public Health	64	(27.6)
Clinical	168	(72.4)
		
Location of Medical Training		
Mozambique (UEM)†	613	(84.8)
Cuba	70	(9.7)
USSR/Bulgaria/E Germany	37	(4.8)
Brazil	1	(0.1)
Portugal	1	(0.1)
		
Work status		
Currently working	693	(95.9)
Studying full-time	12	(1.7)
Not working (due to death)	13	(1.8)
Not working (due to illness)	3	(0.4)
Not working (reason unspecified)	2	(0.3)
		
Main duties of active physicians‡		
Clinical	493	(71.1)
Management	142	(20.5)
Teaching	30	(4.3)
Research	18	(2.6)
Other	5	(0.7)
Unknown	5	(0.7)
		
Geographic location of active physicians‡		
Capital city (Maputo)	342	(49.4)
Province	284	(41.0)
Outside Mozambique	65	(9.4)
Unknown	2	(0.3)

† UEM: Eduardo Mondlane University.

‡ Includes physicians studying full-time.

**Figure 1:**
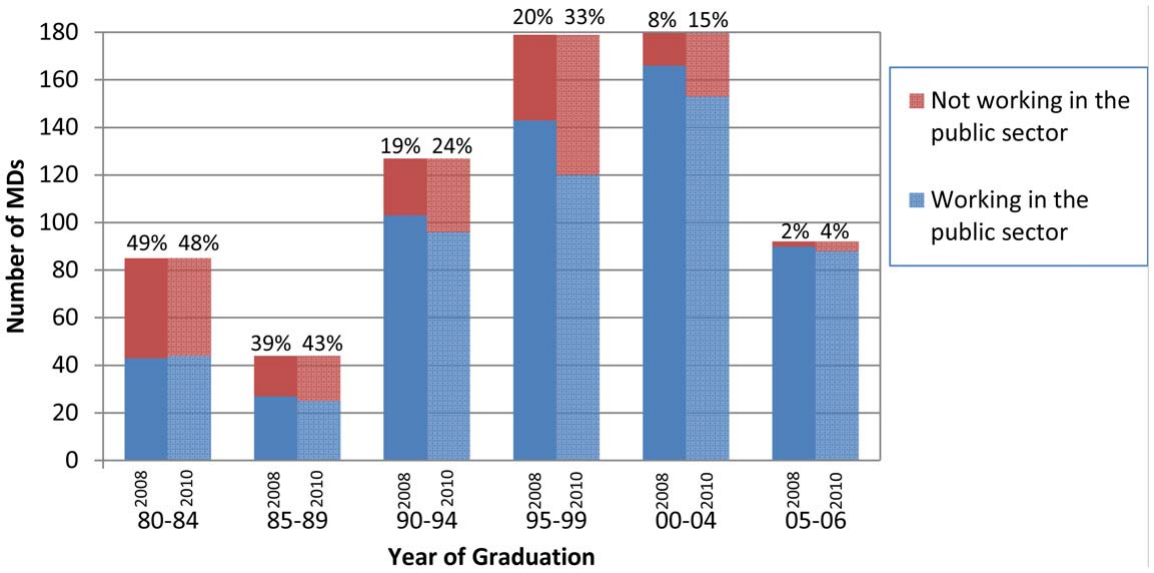
Percentage of physicians working outside the public sector in 05/2008 and 07/2010 by graduation year.

**Figure 2:**
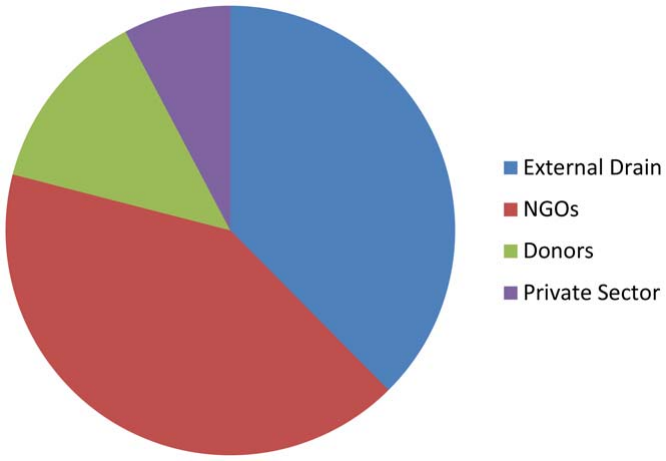
Employment category for MDs working outside the public sector in 07/2010.

Over 25 percent of Mozambican physicians had migrated outside of the public sector by July, 2010 (Table 2). The proportion working outside the public sector increased directly with number of years since medical school graduation (Figure 1). Among physicians who migrated from the public sector, a higher proportion did so for employment within (62.4 percent) than outside Mozambique (37.6 percent), although the proportion of those seeking employment abroad was higher among those graduating medical school between 1980 and 1989 (68.3 percent). Among those leaving the public sector for employment within Mozambique (N = 113), the majority (66.4 percent) worked for NGOs, followed by bilateral and multilateral donors (21.2 percent), and the private sector (12.4 percent) (Figure 2). We estimate that 36.3 percent (N = 41/113) of cases of internal migration had previously held a senior-level management position within the public sector.

**Table 2:** Public sector employment status for Mozambican physicians, by year of graduation.

	Year of Medical School Graduation							
	1980–89		1990–99		2000–06		Total	
	N	(%)	N	(%)	N	(%)	N	(%)
Physicians working outside the public sector (07/2010)	60	(46.5)	90	(29.2)	31	(11.4)	181	(25.5)
Internal migration	19	(31.7)	68	(75.6)	26	(83.9)	113	(62.4)
External migration	41	(68.3)	22	(24.5)	5	(16.1)	68	(37.6)
								
Physicians working outside the public sector (05/2008)	59	(46.5)	60	(19.5)	16	(6.0)	135	(19.0)
Internal migration	19	(32.2)	44	(73.3)	11	(68.8)	74	(54.8)
External migration	40	(67.8)	16	(26.7)	5	(31.3)	61	(45.2)
								
Employment type – cases of internal migration (07/2010)								
NGO	5	(26.3)	49	(72.1)	21	(80.8)	75	(66.4)
Donor	9	(47.4)	11	(16.2)	4	(15.4)	24	(21.2)
Private Sector	5	(26.3)	8	(11.8)	1	(3.9)	14	(12.4)
								
Employment type – cases of internal migration (05/2008)								
NGO	4	(21.1)	30	(68.2)	7	(63.6)	41	(55.4)
Donor	9	(47.4)	9	(20.5)	3	(27.3)	21	(28.4)
Private Sector	6	(31.6)	5	(11.4)	1	(9.1)	12	(16.2)

Based on the experience over 26 months between the 2008 and 2010 data collection periods we estimate an annual incidence of internal and external physician migration to be 3.7 percent. Of the 574 physicians working in the public sector in May, 2008, 6.3 percent (N = 36) left to work for NGOs, 0.5 percent (N = 3) for multi-lateral and bi-lateral donor agencies, 0.5 percent (N = 3) for the private sector, and 1.2 percent (N = 7) for outside Mozambique by July, 2010. Three physicians (N = 2.2 percent) of the 135 working outside the public sector as of May, 2008, returned to work for the public sector. Two physicians (0.4 percent) died during this period. In July, 2010, 59.3 percent of internal migration cases (N = 67/113) worked in the capital city of Maputo. Furthermore, 58.4 percent of internal migration cases (N = 66/113) worked for institutions funded primarily through PEPFAR, compared with 46.0 percent (N = 34/74) in May, 2008.

**Figure 3:**
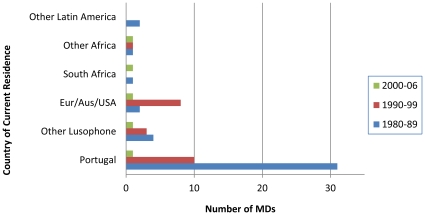
Country of residence by year of graduation for MDs working outside the public sector, 07/2010.

Among Mozambican physicians residing abroad in July, 2010 (N = 68), most were in Portugal (61.8 percent), followed by other high-income countries (16.2 percent), other Portuguese-speaking countries (11.8 percent), and other countries in Africa and Latin America (10.4 percent). Notably, 60.3 percent of physicians living abroad graduated from medical school between 1980 and 1989, of whom 75.6 percent were working in Portugal (Figure 3). Of those working abroad, 69.1 percent were involved in primarily clinical activities, 14.7 percent in management, and 7.4 percent in research (information was not available for 5.9 percent of these physicians).

Among active physicians qualifying before 2003 (N = 520), 69.2 percent (N = 360) were still working in the public sector as of July, 2010, while 18.7 percent (N = 97) had migrated to work for other agencies within Mozambique, and 12.1 percent (N = 63) had migrated outside Mozambique. Those with a post-graduate degree in public health or without a post-graduate degree were at increased risk of working outside the public sector when compared to physicians with a clinical specialty (Table 3). Gender and having qualified from medical training at Eduardo Mondlane University were not significantly associated with working outside the public sector.

**Table 3:** Post-graduate training of physicians graduating before 2003 (N = 520) not working in the public sector in 07/2010.

	N	(%)	Risk Ratio (95% CI)
By post-graduate training			
Clinical specialization	22	(13.4)	-Ref-
No post-graduate training	107	(36.4)	2.73 (1.80 to 4.15)
Public health trained	33	(48.4)	3.61 (2.27 to 5.74)

## Discussion

This study described the magnitude of internal and external migration among Mozambicans physicians qualifying between 1980–2006. We found substantial flight from the public sector, especially among more experienced physicians. Overall, internal migration was a larger contributor than external migration to loss of physicians from the public sector, a difference that accelerated at an increased rate over the 26-month observation period as physicians left in increasing numbers to work in the growing NGO sector. When excluding physicians with stronger ties to the colonial power, Portugal (those qualifying within 10 years after independence), internal migration accounted for more than three times as much physician loss than external migration. Notably, the extent of external migration in this sample does not approximate the high level (over 75 percent) previously reported for Mozambique, which included Portuguese nationals who returned to mainland Portugal at independence [28]. We believe that the level of external migration reported here more accurately represents the effect of human capital flight on the post-independence NHS.

Previous research on brain drain has focused on external migration, generally ignoring migration from the public sector to other institutions in the same country. It has been argued that internal migration does not weaken health systems as physicians largely leave to work for agencies that support the public sector, resulting in better directed aid [29]. We believe that internal migration has a distortive effect on health systems as senior-level managers graduate to well-paying jobs outside the public sector, perpetually leaving junior managers with less training and experience in their place. Given the shortage of physicians, this transition frequently leaves management gaps until replacements are identified, trained and begin their new position. The results of this study support the view that it is the seasoned managers who leave the public sector, including physicians with longer work experience and those with public health training, which aligns with the priorities of international agencies seeking experienced health system managers to provide the inside knowledge and personal connections to meet the policy and implementation expectations of these agencies. This study also found that nearly half of physicians were working in the capital, Maputo, where five percent of the country resides, representing an important impediment to broadly providing quality health services. Given the higher percentage of cases of internal migration residing in the capital city compared with those in the public sector, it is apparent that increased contracting of physicians to work with NGOs and donor institutions does not represent an effective solution to the imbalanced distribution of physicians between urban and rural areas.

An especially worrisome finding of this study is the increased frequency of internal physician migration observed alongside the explosion of the NGO sector in Mozambique, fuelled by the dramatic increase in external assistance from global health initiatives relying on NGOs as the primary channel for aid to meet ambitious targets. There are likely multiple push and pull factors that shape physicians’ decisions to remain within the public sector or pursue outside opportunities. Among important push factors are 1) low salaries, especially for those outside of the capital city who have fewer opportunities to pursue part-time clinical work in the private sector and those with public health training who (with the exception of those with doctoral-level training in public health or a related discipline) are not eligible for an increase in base salary compared with physicians with post graduate clinical specialization; 2) lack of clear career advancement opportunities, particularly among health system managers; and 3) challenging working conditions in a health system with limited resources and great need. Budget ceilings have been highlighted as a factor that contributes to resource constraints in the public sector in Mozambique [30], which may partially explain the rationale for channeling increased external aid through the NGO sector. Though the NGO sector is not a new phenomenon in Mozambique [13], recent funding increases have impacted the sector’s dimensions and relationships with the public sector, and a pluralistic system that brings together NGOs with the public sector will likely continue as long as donors continue to emphasize NGOs as fundamental to their development assistance strategies.

This study has a number of strengths. First, its novel sampling approach provides a comprehensive view of both internal and external migration for physicians trained at all the medical schools that feed medical doctors into Mozambique. Following physicians from training source also captures cases of migration that occur after training but before absorption into the public sector. Our sample also included medical graduates over a 26-year period, providing an indication of how the proportion of physicians leaving the public sector changes along career paths. Finally, by collecting information on employment status at two points over a 26-month period, we were able to describe the impact of increased foreign aid through the NGO sector on migration patterns.

The primary limitation of this study is that key informants were used to develop the list of Mozambican physicians trained outside of Mozambique and to provide information on colleagues’ work status. As a result, the list of physicians trained outside of Mozambique may be incomplete and there may be errors in the classification of the work status and location for physicians in the sample. Because of the relatively high rate of movement in the public sector, misclassification of work status would be expected to lead to underestimates in the degree of physician migration. However, given the extensive triangulation of data from multiple sources, including provincial and hospital human resource records as well as multiple proxy reports, we believe that this bias is unlikely to be substantial and does not greatly affect our conclusions. In addition, we were unable to account for part-time income generation activities outside public sector functions that may be important in explaining why physicians with clinical specialties are less likely to leave the public sector. A second limitation is the focus on physicians, without considering the full range of cadres (such as non-physician clinicians or *técnicos de medicina*, nurses, pharmacists and laboratory technicians) that play management and clinical roles within the public sector, and who are in high demand for employment in the international aid community.

The MOH has taken a number of steps to ameliorate the impact of physician flight from the public sector. It has expanded pre-service training capacity for physicians to replace those who no longer are active in the public sector by increasing medical school class size and by increasing the number of medical schools in the country from one to four (including two additional public universities – Lúrio University in Nampula province and Zambeze University in Tete province – and the private Catholic University of Mozambique in Sofala province). As a result, the annual production capacity for new physician graduates currently exceeds 100, compared with an average of less than 21 from Eduardo Mondlane University in the 25-year period between 1980 and 2004. Workforce expansion has also accelerated for non-physician cadres to substitute clinical and management tasks currently under the responsibility of physicians. Working conditions have improved through strengthened management and logistics systems, as well as health facility rehabilitation. Both across the board and targeted incentives for key management positions have been provided to reduce the salary differential between the public sector and international NGOs and donor agencies. Finally, codes of conduct have been signed with donor agencies and NGOs to limit hiring skilled staff away from the MOH [31], [32].

Despite concrete steps taken by the MOH to stem the flow of workers from the public sector, these efforts have failed prevent the loss of these valuable resources. Ultimately these approaches will not succeed until international agencies take responsibility for their role in contributing to physician migration, and in particular internal migration. Our results indicate that the magnitude of internal migration occurs more frequently than external migration, particularly to the NGO sector, and given the now widely recognized urgency to strengthen struggling public sector health systems and the dramatic increase in resources available through global health initiatives, frank reflection by donors and NGOs is needed on how their hiring practices and projects may undermine the very systems they seek to strengthen.
